# CytoResc – “CytoSorb” Rescue for critically ill patients undergoing the COVID-19 Cytokine Storm: A structured summary of a study protocol for a randomized controlled trial

**DOI:** 10.1186/s13063-020-04501-0

**Published:** 2020-06-26

**Authors:** Helena Stockmann, Theresa Keller, Stefan Büttner, Achim Jörres, Detlef Kindgen-Milles, Julius Valentin Kunz, Josef Leebmann, Claudia Spies, Karl Träger, Sascha Treskatsch, Alexander Uhrig, Carsten Willam, Philipp Enghard, Torsten Slowinski, for the CytoResc Trial Investigators

**Affiliations:** 1 Department of Nephrology and Medical Intensive Care, Charité - Universitätsmedizin Berlin, Berlin, Germany; 2 Institute for Biometry and Clinical Epidemiology, Charité - Universitätsmedizin Berlin, Berlin, Germany; 3 Department of Nephrology, Clinic Aschaffenburg-Alzenau, Aschaffenburg, Germany; 4 Clinic for Nephrology, Transplantation Medicine and Intensive Care Medicine, University Witten/Herdecke Medical Centre, Cologne-Merheim, Germany; 5 Department of Anesthesiology, University Hospital Düsseldorf, Heinrich-Heine-University Duesseldorf, Düsseldorf, Germany; 6 Interdisciplinary Apheresis Center at Passau General Hospital, Passau, Germany; 7 Department of Anesthesiology and Intensive Care Medicine, Charité - Universitätsmedizin Berlin (CCM, CVK), Berlin, Germany; 8 Department of Cardiac Anesthesiology, University Hospital Ulm, Ulm, Germany; 9 Department of Anesthesiology and Intensive Care Medicine, Charité - Universitätsmedizin Berlin (CBF), Berlin, Germany; 10 Department of Internal Medicine/Infectious Diseases and Pulmonary Medicine, Charité - Universitätsmedizin Berlin, Berlin, Germany; 11 Department of Internal Medicine 4-Nephrology and Hypertension, Friedrich-Alexander-University Erlangen-Nürnberg (FAU) and University Hospital Erlangen, Erlangen, Germany

**Keywords:** COVID-19, Randomized controlled trial, protocol, cytokine storm, vasoplegic shock, extracorporeal cytokine elimination

## Abstract

**Objectives:**

Approximately 8 - 10 % of COVID-19 patients present with a serious clinical course and need for hospitalization, 8% of hospitalized patients need ICU-treatment. Currently, no causal therapy is available and treatment is purely supportive. The main reason for death in critically ill patients is acute respiratory failure. However, in a number of patients a severe hyperinflammatory response with excessively elevated proinflammatory cytokines causes vasoplegic shock resistant to vasopressor therapy. A new polystyrene-based hemoadsorber (CytoSorb®, Cytosorbents Inc., New Jersey, USA) has been shown to adsorb effectively cytokines and other middle molecular weight toxins this way reducing their blood concentrations. This has been routinely used in clinical practice in the EU for other conditions where a cytokine storm occurs and an observational study has just been completed on COVID-19 patients. We hypothesized that the extracorporeal elimination of cytokines in critically ill COVID-19 patients with suspected hyperinflammation and shock may stabilize hemodynamics and improve outcome. The primary endpoint is time until resolution of vasoplegic shock, which is a well implemented, clinically relevant endpoint in critical care studies.

**Trial design:**

Phase IIb, multicenter, prospective, open-label, randomized, 1:1 parallel group pilot study comparing the additional use of “CytoSorb” to standard of care without “CytoSorb”.

**Participants:**

Patients are recruited from the Intensive Care Units (ICUs) of 7 participating centers in Germany (approximately 10 ICUs). All patients aged 18- 80 with positive polymerase chain reaction (PCR) test for SARS-CoV-2, a C-reactive protein (CRP) ≥ 100 mg/l, a Procalcitonin (PCT) < 2 ng/l, and suspected cytokine storm defined via a vasoplegic shock (Norepinephrine > 0.2 μg/min/kg to achieve a Mean Arterial Pressure ≥ 65mmHg). Patients are included irrespective of indication for renal replacement therapy. Suspected or proven bacterial cause for vasoplegic shock is a contraindication.

**Intervention and comparator:**

Within 24 hours after meeting the inclusion criteria patients will be randomized to receive either standard of care or standard of care and additional “CytoSorb” therapy via a shaldon catheter for 3-7 days. Filter exchange is done every 24 hours. If patients receive antibiotics, an additional dose of antibiotics is administered after each change of “CytoSorb” filter in order to prevent underdosing due to “CytoSorb” treatment.

**Main outcomes:**

Primary outcome is time to resolution of vasoplegic shock (defined as no need for vasopressors for at least 8 hours in order to sustain a MAP ≥ 65mmHg) in days. Secondary outcomes are 7 day mortality after fulfilling the inclusion criteria, mortality until hospital discharge, Interleukin-6 (IL-6) measurement on day 1 and 3, need for mechanical ventilation, duration of mechanical ventilation, duration of ICU-stay, catecholamine dose on day 1/2/3 after start of “CytoSorb” and acute kidney injury.

**Randomization:**

An electronic randomization will be performed using the study software secuTrial® administered by the Clinical Study Center (CSC) of the Charité – Universitätsmedizin Berlin, Germany. Randomization is done in blocks by 4 stratified by including center.

**Blinding (masking):**

The trial will be non-blinded for the clinicians and patients. The statistician will receive a blinded data set, so that all analyses will be conducted blinded.

**Numbers to be randomized (sample size):**

As this is a pilot study with the goal to examine the feasibility of the study design as well as the intervention effect, no formal sample size calculation was conducted. A total number of approximately 80-100 patients is planned (40-50 patients per group). Safety assessment is done after the inclusion of each 10 patients per randomization group.

**Trial Status:**

Please see the study protocol version from April 24 2020. Recruitment of patients is still pending.

**Trial registration:**

The study was registered on April 27 2020 in the German Registry of Clinical Trials (DRKS) under the number DRKS00021447.

**Full protocol:**

The full protocol is attached as an additional file, accessible from the Trials website (Additional file 1). In the interest in expediting dissemination of this material, the familiar formatting has been eliminated; this Letter serves as a summary of the key elements of the full protocol.

## Supplementary information

**Additional file 1.** Full Study Protocol.

## Data Availability

All individual patient data on which the results of the publication are based will be made available in anonymized form to scientists who present a reasonable analysis plan. The aim is to make the scientific findings available for other research projects. Data requests should be addressed to: torsten.slowinski@charite.de. For data access, the applicant must sign a data access authorization. Furthermore, the study protocol, the statistical analysis plan, the patient information and the patient consent form will be made available to all interested persons. These documents are available on an external website for 5 years. The data will be made available for a total of 3 months up to a maximum of 5 years.

