# Supplementary material for: CytoResc – “CytoSorb” Rescue for critically ill patients undergoing the COVID-19 Cytokine Storm: A structured summary of a study protocol for a randomized controlled trial
Source: Trials. 2020 Jun 26;21:577. doi: 10.1186/s13063-020-04501-0 (PMC7316574; doi:10.1186/s13063-020-04501-0)
Supplement: Supplementary file 1 — Additional file 1. Full Study Protocol. [file 13063_2020_4501_MOESM1_ESM.docx]

CytoResc - CytoSorb Rescue for COVID-19 Cytokine Storm –

Multicenter, prospective, open-label, randomized pilotstudy investigating the effect of the use of CytoSorb in patients with vasoplegic shock due to suspected cytokine storm in patients with COVID-19

**SPONSOR:** Charité - Universitätsmedizin, Berlin

**Representative of the Sponsor:**

Dr. Torsten Slowinski
Charité - Universitätsmedizin Berlin
Medizinische Klinik m.S. Nephrologie
und internistische Intensivmedizin
Augustenburger Platz 1
13351 Berlin

Table of Contents

[1 Protocol Synopsis 4](#_Toc38540389)

[1.1 Title of study 4](#_Toc38540390)

[1.2 Study design: 4](#_Toc38540391)

[1.3 Study centers 4](#_Toc38540392)

[1.4 Study Product 4](#_Toc38540393)

[1.4.1 Reference exposure 4](#_Toc38540394)

[1.4.2 Investigational exposure 4](#_Toc38540395)

[1.5 Study period 4](#_Toc38540396)

[1.6 Study purpose 4](#_Toc38540397)

[1.7 Methods 4](#_Toc38540398)

[1.8 Objectives and Outcome measures 4](#_Toc38540399)

[1.8.1 Primary efficacy endpoint: 4](#_Toc38540400)

[1.8.2 Secondary endpoints: 4](#_Toc38540401)

[1.9 Population 5](#_Toc38540402)

[1.10 Inclusion/Exclusion criteria 5](#_Toc38540403)

[1.10.1 Inclusion criteria: 5](#_Toc38540404)

[1.10.2 Exclusion criteria: 5](#_Toc38540405)

[1.11 Safety assessment 5](#_Toc38540406)

[1.12 Data assessment and Data analysis 5](#_Toc38540407)

[1.13 Sample size 5](#_Toc38540408)

[2 Introduction 5](#_Toc38540409)

[3 Study design 6](#_Toc38540410)

[3.1 Intervention scheme 6](#_Toc38540411)

[3.2 Study Endpoints 7](#_Toc38540412)

[3.2.1 Primary efficacy endpoint: 7](#_Toc38540413)

[3.2.2 Secondary Endpoints: 7](#_Toc38540414)

[3.3 Randomization 7](#_Toc38540415)

[3.4 Blinding assessment 7](#_Toc38540416)

[4 Study assessment 7](#_Toc38540417)

[5 Safety assurance 9](#_Toc38540418)

[5.1 Safety of the procedure 9](#_Toc38540419)

[5.2 Risk mitigation 9](#_Toc38540420)

[5.3 Safety assessment 9](#_Toc38540421)

[5.4 Criteria for Study discontinuation 10](#_Toc38540422)

[6 Data management 10](#_Toc38540423)

[7 Statistical analysis 10](#_Toc38540424)

[8 Ethics and Good Clinical Practice 11](#_Toc38540425)

[8.1 Good Clinical Practice 11](#_Toc38540426)

[8.2 Ethics Committee 11](#_Toc38540427)

[8.3 Informed consent 11](#_Toc38540428)

[8.4 Declaration of Helsinki 11](#_Toc38540429)

[8.5 Data Sharing Statement 12](#_Toc38540430)

[9 Investigator Signature 12](#_Toc38540431)

[10 References 13](#_Toc38540432)

# Protocol Synopsis

## Title of study

CytoResc - CytoSorb Rescue for COVID-19 Cytokine Storm

## Study design:

Multicenter, prospective, open-label, randomized pilotstudy investigating the effect of the use of CytoSorb in patients with vasoplegic shock due to suspected cytokine storm in patients with COVID-19.

## **Study centers**

Approximately 5 sites in Germany with approximately 10 Intensive Care Units (ICU)

## Study Product

CytoSorb (Cytosorbents, Corporation, New Jersey, USA) is a hemoadsorption device containing hemocompatible, porous polymer beads capable of removing cytokines and other medium molecular weight toxins from the blood by size exclusion and surface adsorption. The polymer is both highly adsorptive and biocompatible and has been CE certified in the European Union. Only CE-certified medical devices are used in the study according to their indications. Certificates/Instructions for use are attached

### Reference exposure

Standard treatment without CytoSorb

### Investigational exposure

CytoSorb treatment for 3-7 days initiated within 24 hours after fullfilling the inclusion criteria

## Study period

Cytosorb treatment for 3-7 days

Patient inclusion over 8 weeks

## Study purpose

To evaluate the effect of extracorporeal cytokine elimination with CytoSorb treatment in critically ill COVID-19 patients with suspected cytokine storm.

## **Methods**

This is a randomized, prospective, open-label, multicenter trial investigating the effect of the use of CytoSorb in patients with vasoplegic shock due to suspected cytokine storm in patients with COVID-19. Patients with vasoplegic shock as an indicator of suspected cytokine storm will be randomized to receive either standard of care of standard of care and additional CytoSorb therapy for 3-7 days. Measurement of Interleukin-6 is done on day 1 and 3 respectively.

## Objectives and Outcome measures

### Primary efficacy endpoint:

- time until resolution of vasoplegic shock (defined as no need for vasopressors for at least 8 hours in order to sustain a MAD ≥ 65mmHg) in days

### Secondary endpoints:

- 7 day mortality after fulfilling the inclusion criteria
- mortality until hospital discharge
- measurement of IL-6 on day 1 and 3 of intervention
- need for mechanical ventilation
- duration of mechanical ventilation
- duration of ICU-stay
- catecholamine dose on day 1/2/3 after start of CytoSorb
- acute kidney injury

## Population

The study population will consist of critically ill COVID-19 patients who are consecutively admitted to the ICU of the participating centers and fulfilling the inclusion criteria.

## Inclusion/Exclusion criteria

### Inclusion criteria:

- Males or females aged 18-80 years
- Positive PCR test for SARS-CoV-2
- Vasoplegic shock (Noradrenalin > 0.2 µg/min/kg (aiming MAD ≥ 65mmHg))
- CRP > 100 mg/l
- PCT < 2 ng/l

### Exclusion criteria:

- Patients receiving Tocilizumab (anti-IL6-receptor antibody)
- Patients not applicable for anticoagulation (if Heparin is not applicable local anticoagulation with citrate or systemic Argatroban can be used, severe thrombocytopenia < 20.000/μl is a contraindication
- pregnancy
- bacterial infection

## Safety assessment

CytoSorb is a medical device which is already well implemented in routine clinical practice and CE-certified in the European Union. Several clinical studies and a large registry have not shown any serious side effects. It can be assumed that this can also be transferred to the study participants. The medical device is used within the scope of its approval.

However, in order to exclude risks for the study participants a safety surveillance system will be established including ongoing reports on serious adverse events and mortality sent to the investigators. After each 20 patients an independent Data Safety Monitoring Board (DSMB) will review the safety data of the trial. (see point 5 for details).

## Data assessment and Data analysis

Data capture and data management will be performed using electronic case report forms (eCRF) based on the study software secuTrial^®^ which is provided by the Clinical Study Center (CSC) Charité. Statistical analysis and report writing will be performed when all patients have left the study. Data on mortality and serious adverse events are monitored continuously. (see point 5.)

## Sample size

Not applicable as the study serves as a pilotstudy. Possibly 85-100 patients

# Introduction

Until April 23rd, 2020, 2.640.000 people were tested positive for COVID-19 worldwide. (1, 2) Mortality rates are between 0,7-7%, with variations probably due to different cohorts, different capacities of the health care systems and most likely other unknown factors. Approximately 14 % of the infected patients present with a serious clinical course; 5% need ICU-treatment.(3) This is stretching medical systems around the world to the limit of their capacity and might lead to several non-evidence-based medical treatments. In fact, some organizations are already giving advice to use investigative treatment as compassionate use in COVID-19 patients. Some of those are not without risk. (4)

Until now, treatment is purely supportive. The main cause of death in critically ill patients is acute respiratory failure. (3) There are several observations indicating a cytokine storm leading to acute decompensation of patients which might be a possible option for therapeutic intervention.(5, 6) Several possible treatment options are discussed including streroids, Tocilizumab (an anti-IL6-receptor antibody) as well as extracorporeal elimination. So far, there is no evidence for any of them. On the contrary, steroids and Tocilizumab may even be harmful due to immunosuppression. A recent paper, currently under review for publication in CELL-HOST-MICROBE actually found the expression of proinflammatory genes, especially chemokines, to be markedly elevated in bronchoalveolar lavage fluid of COVID-19 patients as compared to patients with community-aquired pneumonia patients and healthy controls, suggesting that SARS-CoV-2 infection causes hypercytokinemia. (7)

The fact, that multiple chemokines were elevated supports the use of a broader therapy such as extracorporeal cytokine elimination as compared to e.g. Tocilizumab as anti-IL6-receptor antibody.

CytoSorb is an adsorber containing hemocompatible, porous polymere chains, which are able to eliminate cytokines and other toxins of middle molecular weight from the blood. The polymer is highly adsorptive as well as biocompatible and was CE-certified in the European Union.

We hypothesized that the elimination of cytokines might improve outcome in critically ill COVID-19 patients with suspected cytokine storm. As primary endpoint we decided on the time until resolution of vasoplegic shock, a well implemented, clinically relevant endpoint in critical care studies. (8, 9)

Several preclinical studies in animal models showed a reduction of different cytokines and chemokines.(10, 11) Data on clinical effectiveness is inconsistent. In a prospective study in sepsis patients no significant reduction of circulating IL-6 could be detected, however, this might have been due to a heterogeneous patient cohort. (12) Data from an international register suggest clinical effectiveness as well as adequate reduction of cytokine-elimination in a big cohort. (13) In fact, recent data show the succesfull use of CytoSorb in CAR-T-associated cytokinestorm. (14) Despite inconsistent clinical evidence CytoSorb is already broadly used in critically ill patients with sepsis. (13, 15) Apart from the invasiveness of the procedure (there is the need for a big central venous access that most critical patients have anyway) the method is known to be safe. (12, 13, 16)

In order to build prospective reliable evidence (also including negative results) we are planning to do a pragmatic randomized-controlled open-label pilotstudy. The aim of this study is to investigate the effect of the use of CytoSorb in order to eliminate cytokines in critically ill patients with COVID-19.

# Study design

Multicenter, prospective, open-label, randomized pilotstudy investigating the effect of the use of CytoSorb in patients with vasoplegic shock due to suspected cytokine storm in patients with COVID-19. We will consecutively include all eligible patients during a timeframe of eight weeks and randomize them to receive either CytoSorb treatment or standard of care without CytoSorb to eventually plan an adequately powered randomized controlled trial with sample size calculation as a subsequent trial. Study centers will be approximately 5 sites in Germany with approximately 10 Intensive Care Units (ICU). We are assuming an inclusion rate of possibly 85-100 patients.

## Intervention scheme

All patients aged 18-80 tested positive for SARS-CoV-2 via PCR and presenting with vasoplegic shock (defined as need for noradrenaline > 0.2 µg/min/kg (MAD aim ≥ 65mmHg) with infection markers implying a non-bacterial genesis (CRP > 100 mg/l, PCT < 2 ng/l) are eligible for the study. After written informed consent randomization is done using an electronic data system to either receive standard of care with or without CytoSorb treatment. Therapy is done open-label as blinding is not feasible. If not already implemented, patients in the intervention group receive a shaldon catheter after appropriate education. To avoid unnecessary risk of complications, the catheter is inserted by an experienced medical doctor. The intervention is done for 3-7 days irrespectively of indication to renal replacement therapy. Filter exchange is done every 24 hours. If the patient is receiving antibiotics an additional dose is given after every filter exchange in order to control for underdosage. Measurements of IL-6 are done on day 1 and 3 respectively. Primary endpoint is time to resolution of vasoplegic shock. Predefined routine clinical data which serves as outcome variables as well as adverse events are documented in an electronic data base using an eCRF.

Figure 1: Trial Flow Chart

## Study Endpoints

### Primary efficacy endpoint:

- time until resolution of vasoplegic shock (defined as no need for vasopressors for at least 8 hours in order to sustain a MAD ≥ 65mmHg) in days

### Secondary Endpoints:

- 7 day mortality after fulfilling the inclusion criteria
- mortality until hospital discharge
- measurement of IL-6 on day 1 and 3 of intervention
- need for mechanical ventilation
- duration of mechanical ventilation
- duration of ICU-stay
- catecholamine dose on day 1/2/3 after start of CytoSorb
- acute kidney injury

As primary endpoint we decided on the time until resolution of vasoplegic shock, a well implemented, clinically relevant endpoint in critical care. Two big intensive care studies already used this endpoint as secondary endpoint. (8, 9)

## Randomization

An electronic randomization will be performed using the study software secuTrial^®^ admistered by the Clinical Study Center (CSC) of the Charité.

## Blinding assessment

Blinding is not feasible as a clinical intervention is done. The intensive care unit where the patient is treated will be added as random effect in the model in order to control for cluster effects. The statistician will be blinded.

# Study assessment

All laboratory determinations including will be analyzed locally.

Baseline variables:

- Age
- Sex
- Smoker status
- Comorbidities
  - Hypertension
  - Diabetes
  - Coronary heart disease
  - COPD
  - History of malignancy
  - Chronic kidney disease
  - Immunosuppression
  - Other

At inclusion

- Oxygen supplementation
- NIV/High Flow
- Intubation
- ECMO
- Vasoplegic shock
- Dose of Noradrenaline
- Other Vasopressors
- Glucocorticoids
- Antiviral treatment
- SOFA
- APACHE

Outcome parameters

- Time to resolution of shock (d)
- Need for mechanical ventilation
- Days on ventilator after inclusion (d)
- ICU survival
- In-hospital mortality
- 7 day mortality
- Septic shock
- Cardiogenic shock
- Acute kidney injury after Inclusion (AKIN-Stage 1,2,3)
- Dialysis
- Duration of ICU stay
- Vasopressor dose (max) on day 1
- Vasopressor dose (max) on day 2
- Vasopressor dose (max) on day 3
- Change to palliative therapy

Adverse events

- technical failure
- mashine clotting
- other

Serious adverse events

- Cardiac arrest
- Severe bleeding
- Severe allergic reaction
- Severe Complication due to shaldon catheter insertion
- Death
- Other

Complication possibly associated with CytoSorb

- Heparin induced thrombycytopenia Type 2 (HIT 2) if heparine is exclusively needed for CytoSorb therapy
- Complication associated with shaldon catheter insertion if catheter is exclusively needed for CytoSorb therapy
- Severe allergic reaction associated with CytoSorb therapy
- Other

Laboratory Data (day 1+3+7)

- CRP
- Troponin
- D-Dimer
- IL-6 (Only Day 1+3)
- Ferritin
- LDH
- Creatinine
- ALT
- PCT
- Phosphate

# Safety assurance

## Safety of the procedure

Apart from the invasiveness of the procedure (there is the need for a big central venous access that most critical patients need anyway) the method is known to be safe. (12, 13) (15) Possible complications associated with the implementation of the central venous access are bleeding, infection, malpuncture including arterial malpuncture, damage to surrounding organs including lung injuries requiring drainage, thromboses, embolisms and in very rare cases strokes. Rarely allergic reactions to the system are described, (17) which would lead to immediate disruption of the therapy and adequate antiallergic therapy. There is the need for anticoagulation (PTT aim 60-80), which is usually done with Heparine. Heparin induced thrombocytopenia (HIT) is a known adverse events associated with heparin. In suspected or proven HIT patients heparin will be stopped immediately. Alternative anticoagulation (citrate or Argatroban) can be used. For patients with increased risk of bleeding citrate anticoagulation is possible. Since it cannot be excluded that drugs, including antibiotics, are partially removed by the filter, patients receiving antibiotic therapy receive an additional antibiotic dosage after each filter change to avoid underdosage.

## Risk mitigation

The implementation of the central venous catheter is only done by experienced doctors and with ultrasound control. If the patient is receiving antibiotics an additional dose is given after every filter exchange in order to control for underdosage.

## Safety assessment

CytoSorb is a medical device which is already well implemented in routine clinical practice and CE-certified in the European Union. Several clinical studies and also a large registry have not shown any serious side effects. It can be assumed that this can also be transferred to the study participants. The medical device is used within the scope of its approval.

However, in order to exclude risks for the study participants a safety surveillance system will be established and will follow the applicable legislation, standards and SOPs of the CSC Charité. Treating physician visits all patients at least twice a day and any adverse event is documented in the patient file as well as in the eCRF of the electronic database and assessed by the investigators for seriousness. SAE will be sent within 24 hours to the sponsor/medical monitor of the trial. A data safety monitoring board (DSMB) with three external reviewers will review the safety during the trial. It is proposed that the DSMB will meet at regular intervals (after every 10 patients included in each randomization group) via videoconference after start of the trial, to review the safety data of the study. Moreover, members of the DMSB will be informed about adverse events every 2 weeks in between the meetings or immediately in case of the occurrence of unexpected events. In addition, members of the DMSB will be informed on the progress of the trial (in particular recruitment and dropout rate) and will give written recommendations to the funding organization if the study should be discontinued. After the inclusion of 10 patients in each randomization group a report is given to the local ethics committee of the Charité.

## Criteria for Study discontinuation

Stopping rules for the individual patient is patient wish, adverse event (e.g. allergic reaction to CytoSorb, contraindication for anticoagulation, severe thrombocytopenia (<20000/ /μl) or proven bacterial infection during treatment course as we want to see the effect of CytoSorb on suspected cytokine storm and distinguish from other effects leading to vasoplegic shock such as sepsis.

As CytoSorb is already part of routine clinical practice and well known to the users and because of the short trial duration of 8 weeks we did not put restrictions on inclusion rates for each participating site.

Stopping rules for the whole trial are safety concerns due to the ongoing surveillance of mortality and adverse events by investigators as well as the DSMB. Furthermore, an interim analysis of the data is done after the inclusion of 10 patients in each group and a new consultation of the ethics committee is mandatory.

# Data management

Routine clinical data are continuously documented in the patient file. Due to the acute situation and pragmatic study design data collection is restricted to the most important clinical outcome variables. The personal data such as name and date of birth will only be accessible to the attending physician. All protocol‐required information collected during the trial will be documented in an electronic case report form (eCRF) by the investigator, or a designated representative. Data capture and data management will be performed using electronic case report forms (eCRF) based on the study software secuTrial^®^ which is provided by the Clinical Study Center (CSC) Charité. secuTrial^®^ features remote web based data capture and performs authentication procedures, role management, query management and encrypted and secured connections. The data will only be hosted within the Charité IT infrastructure. All modification in the eCRF will be documented by an internal audit trail. Data will be captured shortly after source data documentation and reviewed by programmed plausibility-checks to assure the quality of the data. After the end of the study all data will be exported in an appropriate format and tested by SAS for plausibility and consistency. The database will be closed after accomplished data cleaning. Electronic documentation includes all exported files, SAS scripts, data protocol and the closed database. The participant’s data will be saved and transferred in pseudonymous form. All regulative requirements applying to data protection will be met. Re-identification of a participant’s name is only possible at the study site. The results of the above-mentioned study, compiled according to groups, will be published in a medical journal without the possibility of reference to the respective person and regardless of whether the results are positive or negative. The data recorded outside the medical record will be destroyed by the end of the study.

# Statistical analysis

A recent retrospective observational study showed a mortality of 61.5% in a cohort of 52 critically ill COVID-19 patients. (18) Due to both the explorative character of the study (new virus, heterogeneous data on efficacy of CytoSorb-treatment in general an no data in COVID-19-patients at all) and few data on possible recruiting rates (patient flow not estimable, patient condition not estimable) we cannot make assumptions on the effect size of the intervention. That is why, supported by the Institute of Biometry and Clinical Epidemiology of the Charité, we decided on an investigator-initiated clinical pilotstudy in order to evaluate the feasibility of recruitment and gain knowledge concerning the intervention effect. We will consecutively include all eligible patients for a period of eight weeks and randomize them to either receive CytoSorb treatment or standard of care without CytoSorb in order to plan an adequately powered randomized controlled trial with the gained knowledge on effect size and recruitment feasibility as a subsequent trial.

The primary analysis in order to evaluate the effect of the intervention will be carried out with a shared frailty model with time until resolution of vasoplegic shock as outcome variable censored at the time of death. The randomization group will be included as fixed effect and additionally possible confounder such as age, sex and pre-existing illness.

The intensive care unit where the patient is treated will be added as random effect in the model in order to control for cluster effects.

As an additional analysis, death will be treated as competing risk, results will be shown in a cumulative incidence function.

Secondary endpoints will be evaluated with adequate models (proportional hazards model / ANCOVA / logistic regression) depending on distribution of endpoint variables.

All the analyses will be explorative and will not be controlled for multiple testing. Since missing data is not expected, imputation is not planned.

Blinding of the investigator and patient is not possible as a clinical intervention is done, statistician will receive a blinded data set, so that all analyses will be conducted blinded.

# Ethics and Good Clinical Practice

## Good Clinical Practice

This study must be carried out in compliance with the protocol and the principles of GCP. By signing this protocol, the investigator agrees to adhere to the instructions and procedures described in it and thereby to adhere to the principles of GCP to which it conforms.

## Ethics Committee

This study was approved by the local ethics committee.

## Informed consent

The investigator must explain to each patient the nature of the study, its purpose, the procedures involved, the expected duration, the potential risks and benefits involved and any discomfort it may entail. Each patient must be informed that participation in the study is voluntary and that he/she may withdraw from the study at any time and that withdrawal of consent will not affect his/her subsequent medical treatment or relationship with the treating physician. This informed consent should be given by means of a standard written statement, written in nontechnical language. The patient should read and consider the statement before signing and dating it and should be given a copy of the signed document. If a patient is not able to give written informed consent himself, and a legal representative exists, the consent of the representative will be obtained in advance. For all other patients incapable of informed consent, urgent care will be requested immediately. If the patient regains consciousness during the course of the study, he/she will be informed about the study and can subsequently consent to participate in the study. If the patient does not retrospectively agree with the study, his/her data will be discarded. If the patient does not regain consciousness, the appointed caregiver decides retrospectively on participation in the study. If the patient dies before urgent care could be installed, we would suggest the following procedure: the personal data of the person concerned is processed and evaluated anonymously and in an anonymized manner. In this way, the protection of the personality and the data protection interests of the person concerned are safeguarded. Furthermore, this procedure does not compromise the processing objective of the research project.

## Declaration of Helsinki

The investigator must conduct the trial in accordance with the principles of the Declaration of Helsinki.

## Data Sharing Statement

All individual patient data on which the results of the publication are based will be made available in anonymized form to scientists who present a reasonable analysis plan. The aim is to make the scientific findings available for other research projects. Data requests should be addressed to: torsten.slowinski@charite.de. For data access, the applicant must sign a data access authorization. Furthermore, the study protocol, the statistical analysis plan, the patient information and the patient consent form will be made available to all interested persons. These documents are available on an external website for 5 years. The data will be made available for a total of 3 months up to a maximum of 5 years.

# Investigator Signature

I confirm that I have read this protocol and agree to conduct this study in accordance with all stipulations of the protocol and in accordance with ICH guidelines, GCP, and applicable local requirements.

**Investigator**

Date: ________

Signature: _________________________

Name (block letters): ______________________________

# References

1. Dong E, Du H, Gardner L. An interactive web-based dashboard to track COVID-19 in real time. The Lancet Infectious Diseases.

2. JohnsHopkinsCoronavirusResourceCenter. 2020 [Available from: <https://coronavirus.jhu.edu/map.html>.

3. Ruan Q, Yang K, Wang W, Jiang L, Song J. Clinical predictors of mortality due to COVID-19 based on an analysis of data of 150 patients from Wuhan, China. Intensive Care Med. 2020.

4. Brescia-Renal-Covid-Task-Force. Management of patients on dialysis or with a kidney transplant during COVID-19 infection 2020 [Available from: <https://www.era-edta.org/en/wp-content/uploads/2020/03/COVID_guidelines_finale_eng-GB.pdf>).

5. Ronco C, Reis T, De Rosa S. Coronavirus Epidemic and Extracorporeal Therapies in Intensive Care: si vis pacem para bellum. Blood Purif. 2020:1-4.

6. Mehta P, McAuley DF, Brown M, Sanchez E, Tattersall RS, Manson JJ. COVID-19: consider cytokine storm syndromes and immunosuppression. Lancet. 2020.

7. Zhou ZaR, Lili and Zhang, Li and Zhong, Jiaxin and Xiao, Yan and Jia, Zhilong and Guo, Li and Yang, Jing and Wang, Chun and Jiang, Shuai and Yang, Donghong and Zhang, Guoliang and Li, Hongru and Chen, Fuhui and Xu, Yu and Chen, Mingwei and Gao, Zhancheng and Yang, Jian and Dong, Jie and Liu, Bo and Zhang, Xiannian and Wang, Weidong and He, Kunlun and Jin, Qi and Li, Mingkun and Wang, Jianwei. Overly Exuberant Innate Immune Response to SARS-CoV-2 Infection. . CELL-HOST-MICROBE-D-20-00205 2020.

8. Venkatesh B, Finfer S, Cohen J, Rajbhandari D, Arabi Y, Bellomo R, et al. Adjunctive Glucocorticoid Therapy in Patients with Septic Shock. N Engl J Med. 2018;378(9):797-808.

9. Annane D, Renault A, Brun-Buisson C, Megarbane B, Quenot JP, Siami S, et al. Hydrocortisone plus Fludrocortisone for Adults with Septic Shock. N Engl J Med. 2018;378(9):809-18.

10. Hosgood SA, Moore T, Kleverlaan T, Adams T, Nicholson ML. Haemoadsorption reduces the inflammatory response and improves blood flow during ex vivo renal perfusion in an experimental model. J Transl Med. 2017;15(1):216.

11. Peng ZY, Carter MJ, Kellum JA. Effects of hemoadsorption on cytokine removal and short-term survival in septic rats. Crit Care Med. 2008;36(5):1573-7.

12. Schädler D, Pausch C, Heise D, Meier-Hellmann A, Brederlau J, Weiler N, et al. The effect of a novel extracorporeal cytokine hemoadsorption device on IL-6 elimination in septic patients: A randomized controlled trial. PLoS One. 2017;12(10):e0187015.

13. Friesecke S, Träger K, Schittek GA, Molnar Z, Bach F, Kogelmann K, et al. International registry on the use of the CytoSorb® adsorber in ICU patients : Study protocol and preliminary results. Med Klin Intensivmed Notfmed. 2019;114(8):699-707.

14. Stahl K, Schmidt BMW, Hoeper MM, Skripuletz T, Möhn N, Beutel G, et al. Extracorporeal cytokine removal in severe CAR-T cell associated cytokine release syndrome. J Crit Care. 2020;57:124-9.

15. Ankawi G, Xie Y, Yang B, Xie Y, Xie P, Ronco C. What Have We Learned about the Use of Cytosorb Adsorption Columns? Blood Purif. 2019;48(3):196-202.

16. Friesecke S, Stecher SS, Gross S, Felix SB, Nierhaus A. Extracorporeal cytokine elimination as rescue therapy in refractory septic shock: a prospective single-center study. J Artif Organs. 2017;20(3):252-9.

17. CytoSorbentsEuropeGmbH. 2020 [Available from: <https://www.cytosorb-registry.org/wp-content/uploads/2014/08/CytoSorb-Gebrauchsanleitung.pdf>.

18. Yang X, Yu Y, Xu J, Shu H, Xia J, Liu H, et al. Clinical course and outcomes of critically ill patients with SARS-CoV-2 pneumonia in Wuhan, China: a single-centered, retrospective, observational study. Lancet Respir Med. 2020.
